# Community-based dengue vector control: experiences in behavior change in Metropolitan Manila, Philippines

**DOI:** 10.1179/2047773212Y.0000000061

**Published:** 2012-12

**Authors:** Fe Espino, Jesusa Marco, Nelia P Salazar, Ferdinand Salazar, Ysadora Mendoza, Aldwin Velazco

**Affiliations:** 1 Research Institute for Tropical Medicine, Filinvest, Alabang, Muntinlupa City, Philippines; 2 De La Salle University, Taft Avenue, Manila, Philippiners

**Keywords:** Behaviour change, Community participation, Dengue control, Philippines, Urban

## Abstract

Dengue is the most important mosquito-borne disease in the Philippines, especially in Metropolitan Manila where communities are socially and economically diverse, and city governments struggle to provide basic services such as continuously available, piped water supply to residents. We examined responses to introducing water container management to control dengue vectors in two diverse communities in Masagana City: Village A (gated community) and Village B (informal settlers community). The roll out of the intervention was carried out by the study team, dengue control personnel and local health workers (BHWs). A behavioural change framework was used to describe the community responses to the introduction of a new vector control intervention - household water container management. Although, the desired outcome was not achieved during the study's timeline, observation on processes of behaviour change underscored the importance of understanding the social nature of the urban communities, often overlooked structures when dengue control program and researchers introduce new dengue control interventions.

## Introduction and rationale

The number of dengue and severe dengue cases reported from sentinel sites in the Philippines increased from 37, 101 in 2006 to 118, 868 in 2010.^1^ The disease is reported throughout the year, with a dramatic rise following the onset of rains. Although once confined to urban and semi-urban areas, dengue is now widespread in rural areas of the Phillippine Archipelago. It has now become the most important vector-borne disease in the country.

Dengue control can be effectively addressed with community involvement. It is a responsibility shared by many, and inter-sectoral cooperation is a strategic approach for successful interventions.^2^–^5^ Community-based programmes have aimed at modifying health-risk behaviours and the conditions that produce and support them. These programmes have included community-wide health education, risk factor intervention, and efforts designed to change laws or regulatory policy in areas where health is affected. These undertakings rely on community organization techniques to boost community leadership and resources, and to plan interventions.^6^ Most community-based programmes for dengue control have focused on eliminating domestic repositories of the mosquito vector and ensuring that they are free of *Aedes spp*. Community participation research has concentrated on neighbourhoods which are subject to intervention and/or are vulnerable communities; such research has not considered groups who implement and could sustain these interventions, or are responsible for disease control.^7^ Societal sectors that have some interest in, or responsibility for the control of dengue have been identified: national control programs, local governments, environment and urban planning, education, science and technology, the media, the private sector, and communities in endemic areas.^5^,^8^,^9^ These stakeholders at community level (e.g. households, primary health care workers) need to interact with technical officers at the local government level (e.g. sanitation inspectors) in order to create positive effects.

The ecological and social factors underlying the success of disease control necessitate thinking beyond the realm of vector-host-infectious agent interrelationships.^10^ Knowledge alone does not influence the practice of anti-dengue measures, but lifestyle affects the proliferation of mosquito vectors.^11^ Dengue control, therefore, should include cultivating attitudinal and behavioral change, and develop new competencies among health providers and the community.^12^,^13^ Simultaneously, local governments and national vector-borne disease control programmes must risk departing from traditional expectations of community participation.^7^ In the milieu of highly urban communities of diverse social groups, politically polarized local governments, inadequate basic water services creating the need to store water and, thus, dengue vector breeding, how can household participation in new dengue control strategies be initiated and sustained? The persistant problem of inadequate water supply forces households to store water throughout the year. Provision of this service, in addition to solid waste management, are important external factors to sustain community-based vector control.^14^

Masagana City^1^ was the site of a two-phase study to determine the ecosystem of dengue vector density, identify possible interventions, and design locally appropriate approaches to reduce vector density. The findings of the first phase have been published.^15^ Based on pupa surveys conducted during the rainy (2007) and dry (2008) seasons, periodic and focal strategies aimed at epidemiological key containers would reduce the number of vector breeding sites considerably.^16^ In 2008, the city government passed an ordinance that recognized the local government’s responsibility in community-based dengue control,^2^ stipulated the creation of a *barangay*^3^ counterpart to the city dengue task force called the *barangay* dengue watch, and evoked elimination of vector breeding sites through the container management by the imposition of penalties and fines. However, at the time of writing, implementing rules and regulation of these ordinances have yet to be written.

**Figure 1:**
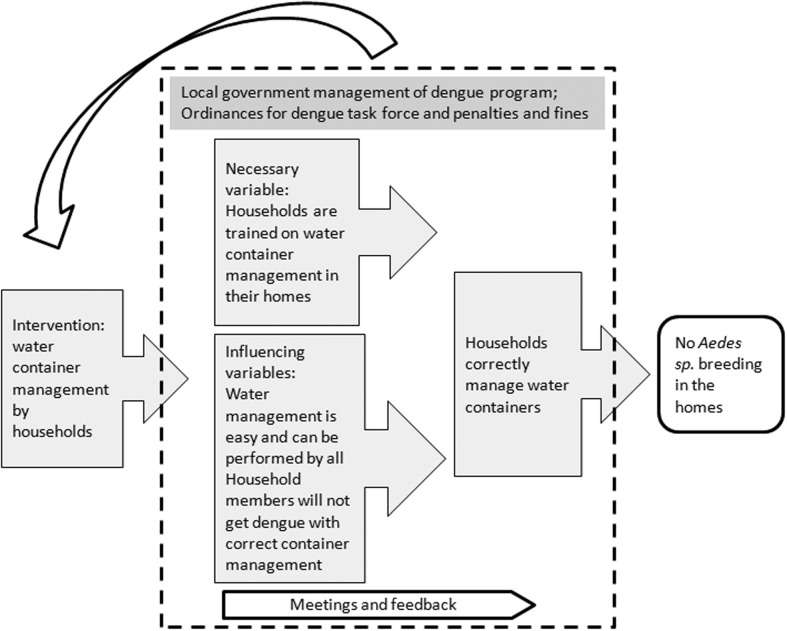
Framework for household water management in Masagana City, Philippines.

This paper describes the processes in setting up an intervention aimed at mobilizing households to regularly inspect water containers as a strategy to reduce vector breeding sites of *Aedes sp*. and operating within a devolved vector-borne disease control programme. We assessed this process following a behavior change framework for household container management shown in Figure 1. We believe that the health belief model by itself is not sufficient and that other factors (e.g., social and political environment) are needed to explain community responses to new dengue vector control interventions. Village A (gated community) and Village B (informal settlers community) are presented to illustrate and contrast the processes and community responses in rolling out the intervention in two socio-economic diverse urban communities.

## Methods

### Description of study sites

Village A and Village B were selected from among 12 randomly-selected clusters.^15^ These two villages differed in mean number of years of formal schooling of residents, housing condition, open spaces and green areas. Village A was better than Village B in these characteristics. Storing water for domestic use was a common practice. Homes in Village A had piped water from a deep well fitted with a motorized pump, and water was often brought in by trucks. In Village B, residents collected water from a common source. Overhead tanks, drums, pails, basins, and jars were the most common types of containers used.

### Intervention roll out and community feedback

Water container management by households was introduced during a series of meetings and workshops held with local government and health officials, *barangay* captains and homeowners’ association presidents.^4^ The city sanitation inspectors, *barangay* officials and community health workers (called *barangay* health workers or BHWs) were trained to teach household members to inspect containers for immature forms of mosquitoes and manage the containers when these were found (i.e. discard the contents and clean the containers). The dengue task force responsibility to oversee households carrying out this task, particularly during the rainy season, was emphasized. An instructional guide on regular container inspection and management was provided to households and BHWs to monitor household compliance with the instructional guide. Process, progress, and outcome indicators for management of water containers at household, *barangay* and city levels were drawn up before the roll out. A checklist was developed by the study team for household members to use to guide regular water container inspection. This form was monitored and collected by BHWs during monthly visits to households.

Group discussions were held six months after the roll out of the intervention. Households’ impression, difficulties in compliance with the task, and suggestions for improvement were discussed. Likewise, BHWs and sanitation inspectors shared their experiences in implementing the strategies through FGDs and interviews respectively. Monitoring the management of household water containers through direct observation continued for three months. Community responses to the intervention were documented through field notes, minutes of meetings, group discussions, and interviews.

### Framework for household water management

To facilitate our understanding of community responses to a new intervention, the framework we used combined two models of behavior change for the desired outcome (absence of *Aedes sp*. breeding in the homes): the health belief model and the process approaches model.^17^ Residents in Masagana considered dengue a serious and preventable disease.^15^ We, therefore, assumed that people in Masagana city believed that managing water containers in their homes will prevent them from contracting dengue. We added variables described as necessary for (intention, skills and absence of environmental constraints) and influencing (positive attitude, normative pressure to perform, consistency with self-image, positive emotional reaction, and self-efficacy) behavioral change.^18^ In household container management context, these are, respectively, households that are trained on home water management, and where water management is easy to do and can be performed by all household members. The process approaches model was also included. In this model, the primary goal is ‘to correctly move through a normative process and thus produce significant behavior change.’^17^ Our assumption was that a trained household member will correctly manage water containers. Interventions that included intermediate-structural determinants (programs, practices, and laws and policies) in their framework had positive impact on behavior change to reduce disease incidence of HIV/AIDS among urban youth.^19^ These determinants in Masagana City were local government management of dengue control and ordinances that created a dengue task force and penalties for households where Aedes spp. breed. These are illustrated in Figure 1.

## Data management and analysis

Interviews and focus group discussions were recorded and transcribed. Field notes were shared and reviewed during regular study team meetings. Minutes were made of meetings, and workshops activities and outputs were documented. These transcriptions and documents were encoded and categorized using AtlasTi^TM^ (Scientific Software Development GmbH, Berlin, September 2009).

The study protocol, research participant information sheet, and written informed consent form received a written, favourable approval from the Institutional Review Board of the Research Institute for Tropical Medicine, Department of Health, Philippines, and the Ethics Committee of the UNICEF/UNDP/World Bank/WHO Special Programme for Research and Training in Tropical Diseases. Written informed consent was obtained from all household heads for their involvement in research activities. A list of households in the 12 clusters in phase I was obtained from either the homeowners’ association of the gated community, or the *barangay* of the cluster. The names written on the list were considered as the household head. Written informed consent was also obtained from local officials and health workers for interviews and focus group discussions.

## Results

### Setting up and monitoring water container management

#### Village A (gated community)

It took several attempts by the research team to schedule a roll-out meeting with the *barangay* captain under whose jurisdiction Village A belonged. The training session for BHWs and the other health personnel was held in the *barangay* office. *Barangay* officials were invited to participate but no one accepted. Likewise, only eight of the 101 households in Village A attended the scheduled training session. They were household help and not family members. Due to this poor turnout, it was decided that trainings be conducted in each household. The homeowners’ association officers, the *barangay* health centre physician, two sanitation inspectors, and eight BHWs organized themselves as the Village A dengue task force. The BHWs formed themselves into groups of two to train the households and were supervised by the sanitation inspectors. The household training visits began after a month. 84 households were visited in two weeks. The study team accompanied the task force for the first 15 households only. Over the next two months, visits were made to each household at least once. The BHWs, unfamiliar in dealing with gated communities, encountered difficulties. The addresses of some households could not be located or no one was at home. Some households refused to allow the BHW to enter into their premises, citing security reasons or that the household head was at work. Others were annoyed by the visits and asked when they would end. One of the BHWs mentioned that it was hard to teach people in Village A^5^ compared to informal settler communities in Masagana City. By the third month, 19 (22.2%) households had refused the BHW visits and were considered to have withdrawn their consent to participate. Despite this, the BHWs continued their work. They patiently reminded households of the benefits of water container management. They perceived that their visits were slowly being appreciated because the information they were giving to households were also heard broadcasted on popular television shows. One BHW said that a household member in Village A told her, ‘*It is good that we are being visited by you and that there is a project like this*.’

A member of the Village A dengue task force observed that households required constant supervision from the BHWs. This person also said that the project intervention had made homeowners more aware of inspecting and covering their water containers. Unfortunately, no household in Village A responded to the invitation to participate discussions regarding the intervention despite encouragement from the homeowners’ association. It was later learned that there were misunderstandings and conflicts (not related to the study) between the homeowners’ association officers and residents in Village A.

#### Village B (informal settlers community)

*‘We feel safe and secure from dengue.’* (Housewife, Village B)

Due to the poor attendance of the training session in Village A, it was decided that house visits would also be carried out in Village B. Meetings were held with *barangay* officials to encourage them to organize themselves as *barangay* dengue watch in order to supervise household water container management. The training session was held in the *barangay* office, and was attended by the health centre physician, two sanitation inspectors, four BHWs, and a *barangay* councillor. Ninety-nine household names were distributed among the BHWs. One sanitation inspector supervised two BHWs. The study team did not join the BHWs during the house-to-house visits. The BHWs assigned to Village B remarked that the householders were hospitable and this was perhaps due to the fact that one of the BHW resided in the area. The BHWs also found households easy to instruct regarding water container management. They observed that a household was more cooperative if the family member or a close relative had dengue.

86 of the targeted 99 households in the cluster consented to participate and only seven households (7%) withdrew this consent at the end of three months. Participants in group discussions said that the visits made residents more aware of dengue control, which, they believed, lowered the number of dengue cases in the area. They considered households who did not inspect their containers as lazy and with no concern for dengue control. These experiences were discussed during Village B informal discussions with community residents that take place in public areas. Residents observed that the sanitation inspectors were carrying out more health education on dengue than in previous years. Inspecting water containers for mosquito larvae and pupae made them feel safe and secure from dengue.

### Political leadership and dengue control –who’s in charge?

During the early part of intervention roll out, a meeting was held with the mayor to present phase I results, discuss the proposed intervention and solicit his support. He disclosed that his wife headed an organization with dengue control in its agenda. There were failed attempts for the study team to meet again with the mayor to discuss the city dengue task force and met instead with one of his assistants. During this meeting he stressed that dengue control was a priority of the city government. He added that the dengue task force members had to be frequently reminded of their responsibilities regarding dengue control. It was learned from the interview with the city health officer that because dengue control was their responsibility, the honorarium task force members had stopped. Since its creation in 2008, the task force had two meetings. A meeting with the task force was not fruitful. Only a few members attended; others sent representatives from their office. Although the sanitation inspectors suggested that it was better to involve the *barangay* officials because of their resources to supervise household activities, a member of the city task force observed that there was not much involvement of *barangay* officials in dengue control. During the entire study period, only one of the nine Masagana city *barangay*s set up its ‘*barangay* dengue watch’ despite the city health officer’s lectures to *barangay* officials on dengue control.

## Discussion and conclusion

The targeted behavior change (outcome indicator of this study) particularly at the household level (i.e., management of water containers) was not achieved during this project lifetime. We commenced this project with the health belief model for behavior change. Households would manage water containers after they received training. The task was expected to be made easier with the instruction guide provided, and performed correctly with supervision from the dengue task force (the BHWs). Further, positive influence from community perceptions of dengue and the benefits of disease control was anticipated. The intermediate-structural determinants existed in Masagana city for residents to participate in dengue vector control and the local government to fulfill its responsibilities. Local ordinances for dengue task force and water container management did not guarantee effective enforcement mechanisms, and implementing rules and/or regulations were absent. Also there were conflicting views from the local players (mayor’s assistant, member of the dengue task force, *barangay* official) as to whom the responsibility of dengue control in the city belonged. It was expected that the city’s dengue task force, would organize and plan control-related activities, but the meeting with this group and other local government officials, health personnel and *barangay* workers disclosed the absence of concrete leadership within the health and political sectors.

There was evidence of community participation in Masagana city. The BHWs of Village A (who were all women) revealed that their training and monitoring visits to the households has given them personal fulfillment and they were philosophical about their experiences. The persistence of these women despite the difficulties they encountered, the on-the-spot formation of a *barangay* task force for Village A and ‘bench conferences’ in Village B are other examples. The process indicators that are shaped by variables such as leadership, planning and management, women’s involvement, and monitoring and evaluation were probably more suitable to capture relevant information in community-based control programs than the final outcome indicators such as the reduction of vector densities or viral transmission.^20^

Our behavior change model had focused on the behavioral outcome of the intervention, and we overlooked a requisite behavior in the process of obtaining the desired behavior: households allow health care workers into their homes to train them on container management. This determined BHWs’ effectiveness in introducing the intervention. In Village B, the BHWs and sanitation inspectors had more access to, and influence on the households than in Village A. In addition, the BHWs were either residents of Village B or the *barangay* where the village was located. Although the officers of the homeowners’ association of Village A were receptive to the strategy, it was difficult to involve residents in water container management. This was evident from the low attendance to the training session. The investigators and the study team were identified with the homeowners’ association in Village A; this may have had a negative impression as was shown by their absence from the FGD sessions. Even before the intervention was rolled out, attendance to homeowners’ association meetings was already poor. Further, there were a significantly higher number of households in Village A who withdrew their consent at the end of three months compared with Village B. A question to address for communities of this nature is: who is a suitable champion for strategies in dengue control?

Local government support and a well-functioning control program have been regarded as catalytic in the success of new interventions for dengue.^4^,^21^ Vector control programmes are devolved in the Philippines but local governments struggle with this responsibility.^22^ Local government leadership in this regard was lacking in Masagana City. The political environment, social and economic differences in the two urban communities, and their take up of new dengue control strategies may affect any attempt at introducing new interventions for dengue control.

In retrospect, we should have adopted a strategy that negotiated socially responsible leadership from the Masagana city government, households in Village A and homeowners’ association. It has been shown in Cambodia that health education materials and strategies have to be practical, and their messages consider actual conditions of communities that are recipients of the materials.^3^ In a similar context, when introducing dengue vector control strategies, including to communities participating in research, approaches need to be adaptable. As shown in Masagana city, community responses of Village A and B were as varied as their social and environmental characteristics and community priorities. Engaging communities of different social and economic backgrounds to participate in dengue vector control activities is a skill that the Philippines’ dengue control program lacks.^23^ The investigators took on the role of stakeholder for some time, and witnessed the potential strengths and numerous limitations which have to be overcome. Technocrats and scientists in the national agencies must take this risk to know and understand the values that local stakeholders can bring to the table. This perspective needs to be integrated into the framework of behavioral models in dengue and other vector-borne disease control. The key is to understand the context of the behavior and to respond adequately.^24^

## Footnotes

1 The names of the city and villages have been made anonymous.

2 The Philippines’ Local Government Code was passed in 1993.

3 The *barangay* is the smallest geo-political unit in the Philippines. The elected officials directly.

4 Homeowners association in gated communities is the counterpart of *barangay* councils. Officials are elected but they cannot create ordinances.

5 In another gated community included in this study, a household member told the BHW “we have a doctor who can explain (dengue control) better than you.”
